# Integrative Transcriptomics Uncovers IFN-β Signature and IFITM3 as Putative Molecular Mediator in MS

**DOI:** 10.3390/ijms27125329

**Published:** 2026-06-12

**Authors:** Alessandro Maglione, Rachele Rosso, Simona Rolla, Eleonora Virgilio, Marinella Clerico

**Affiliations:** 1 Department of Computer Science, University of Turin, 10124 Torino, Italy; 2 Department of Clinical and Biological Sciences, University of Turin, 10043 Orbassano, Italy; rachele.rosso@unito.it (R.R.); simona.rolla@unito.it (S.R.); eleonora.virgilio@unito.it (E.V.); marinella.clerico@unito.it (M.C.); 3 Neurology Unit, San Luigi Gonzaga Hospital, 10043 Orbassano, Italy

**Keywords:** multiple sclerosis, interferon-beta, transcriptomics, EBV, biomarkers

## Abstract

Neuroinflammation in multiple sclerosis (MS) is driven by the infiltration of myelin-reactive T cells into the central nervous system (CNS). Interferon-β (IFN-β) is one of the earliest disease-modifying treatments (DMTs) approved for MS and remains widely used in special populations (pregnant and elderly patients) owing to its favorable safety profile. However, the exact mechanism of action of this drug and reliable biomarkers of treatment response remain unclear. Transcriptomic profiling and data integration approaches offer powerful tools for investigating complex patterns of regulation and molecular mechanisms underlying therapeutic efficacy. In this study, we performed an integrative analysis of openly available transcriptomic datasets to characterize IFN-β-induced gene expression changes in MS patients. By combining data from large independent cohorts, we identified a 43-gene transcriptional signature consistently associated with IFN-β treatment across disease stages, including progressive MS. To explore the relevance of this signature, we cross-referenced the 43-gene signature with publicly available expression quantitative trait loci (eQTL) datasets to determine whether these genes could be influenced by known MS-associated risk variants highlighting Interferon-Induced Transmembrane Protein 3 (IFITM3) as a candidate molecular mediator of MS. This integrative approach provides new insights into IFN-β-driven immune modulation and supports the development of therapeutic strategies for MS.

## 1. Introduction

Interferon-β (IFN-β) was among the first disease-modifying therapies (DMTs) proposed and approved for multiple sclerosis (MS). Although a progressive shift toward early escalation with high-efficacy DMTs is occurring [1], IFN-β remains used [2,3], owing to its favorable risk–benefit profile [3]. Its use is particularly relevant in low-income countries, and in special populations, such as during pregnancy, in late-onset MS (LOMS), and elderly patients [4,5,6,7]. Moreover, for safety reasons, IFN-β represents an appropriate de-escalation strategy in older patients who have been on long-term high-efficacy DMTs [8,9,10,11].

While the exact mechanisms of action of IFN-β are not fully understood, it primarily affects immunomodulatory and anti-inflammatory pathways in MS patients [12]. IFN-β down-regulates class II major histocompatibility complex molecules on antigen presenting cells, including dendritic cells and B lymphocytes [13], induces interleukin (IL)-10 production in T cells [13,14], and inhibits T-cell migration as a result of blockade of metalloproteases and adhesion molecules [13,15,16]. Moreover, it promotes apoptosis of autoreactive T cells, facilitates the induction of regulatory T (Treg) cells and B cell apoptosis [17,18].

In patients with relapsing–remitting MS (RRMS), lower baseline proportions of CD19^+^ B cells and higher frequencies of CD8^+^ perforin^+^ T cells have been associated with an optimal therapeutic response and achieving no evidence of disease activity (NEDA) [19]. Early activation of interferon-stimulated genes (ISGs), including MX Dynamin Like GTPase 1 (MX1), MX2, XIAP Associated Factor 1 (XAF1), and Lysosome-associated membrane glycoprotein 3 (LAMP3), within the first month of treatment reflects effective biological responsiveness and correlates with improved clinical outcomes [20].

At the cellular signaling level, MS responders display distinct activation patterns of the Janus Kinase (JAK)–Signal Transducer and Activator of Transcription (STAT) pathway in monocytes, characterized by reduced IFN alpha receptor 1 (IFNAR1) and increased IFNAR2 expression following IFN-β stimulation, together with differential STAT1/STAT2 phosphorylation [21]. Immunoregulatory features also appear to influence treatment efficacy. Non-responders tend to exhibit reduced circulating naïve Treg cells, increased terminally differentiated effector memory CD4^+^ T cells, and a higher prevalence of Human Leukocyte Antigen (HLA)-DQ8/DQ2 alleles [22]. In parallel, pharmacogenetic studies have identified polymorphisms in genes such as Cathepsin S (CTSS) and TNF Receptor Superfamily Member 10a (TNFRSF10A) that are associated with long-term clinical stability [12]. Moreover, baseline transcriptomic and proteomic profiles characterized by activation of pro-inflammatory pathways, including Phosphoinositide 3-kinase gamma (PI3K-γ) and Nuclear Factor kappa-light-chain-enhancer of activated B cells (NF-κB) signaling, have been linked to the formation of anti-drug neutralizing antibodies and subsequent poor clinical response [23]. Although many ISGs have been identified over the years [24,25,26], the IFN-β signature has mainly been described in peripheral blood, with insufficient power to predict treatment response. In recent years, increasing evidence suggests that several immunological and molecular biomarkers may help predict responsiveness to IFN-β therapy across different phenotypes of MS.

Importantly, accumulating evidence indicates that endogenous interferon signaling is intrinsically altered in MS, with patients exhibiting reduced baseline serum IFN levels [27,28]. In addition, recent high-throughput genetic screening has identified multiple genetic risk variants near immunologically relevant genes, including ISGs, suggesting a genetic dysfunction of the IFNs pathway in MS [29].

Interestingly, the type I IFNs pathway is highly interconnected with viral response [30]. Most viruses, including herpes viruses, stimulate innate immune response during primary infection predominantly by activating Toll-Like Receptors (TLRs) to induce type I IFNs [31]. In the last few years, Epstein–Barr virus (EBV) has been strongly connected with MS risk, and to date, EBV is associated with higher risk compared to any other known risk factor for MS, suggesting that EBV could be the leading cause of MS disease [32].

Our study aimed to explore the IFN-β-induced transcriptional activity in MS and gain insights on the role of ISGs as molecular mediators of MS disease.

To explore the relevance of this signature, we used available expression quantitative trait loci (eQTL) datasets to determine whether these genes could be influenced by known MS-associated risk variants.

This characterization may help identify reliable therapeutic biomarkers and clarify the mechanisms associated with IFN-β therapy and the role of ISGs in MS.

## 2. Results

### 2.1. Integrated PBMC Analysis Reveals IFN-β Signature in MS

Three publicly available transcriptomic datasets were included to investigate the transcriptional effects of IFN-β treatment across different clinical forms of MS: GSE16214 [25], GSE73608 [33] and GSE41850 [26] (Supplementary Table S1).

The datasets GSE16214 and GSE73608 comprised peripheral blood mononuclear cell (PBMC) gene expression profiles from RRMS patients. Overall, these cohorts included RRMS patients (*n* = 94 treated, *n* = 82 untreated), treated with IFN-β for 3 months. These datasets were primarily used to characterize the early transcriptional response to IFN-β. To extend the analysis to progressive MS, we additionally considered the SPMS cohort included in GSE73608, which consisted of secondary progressive MS (SPMS) patients (*n* = 25 treated, *n* = 49 untreated), receiving IFN-β treatment for 2 years. This dataset was specifically used to evaluate whether IFN-β-associated transcriptional signatures are preserved in the progressive stage of MS.

Finally, GSE41850 included whole-blood transcriptomic profiles from a mixed cohort of clinically isolated syndrome (CIS), RRMS, and SPMS patients collected after IFN-β treatment (*n* = 106 treated, *n* = 89 untreated). This dataset was used as an independent validation cohort to assess the consistency of IFN-β-induced transcriptional changes across heterogeneous MS phenotypes and blood-derived sample types.

**Figure 1:**
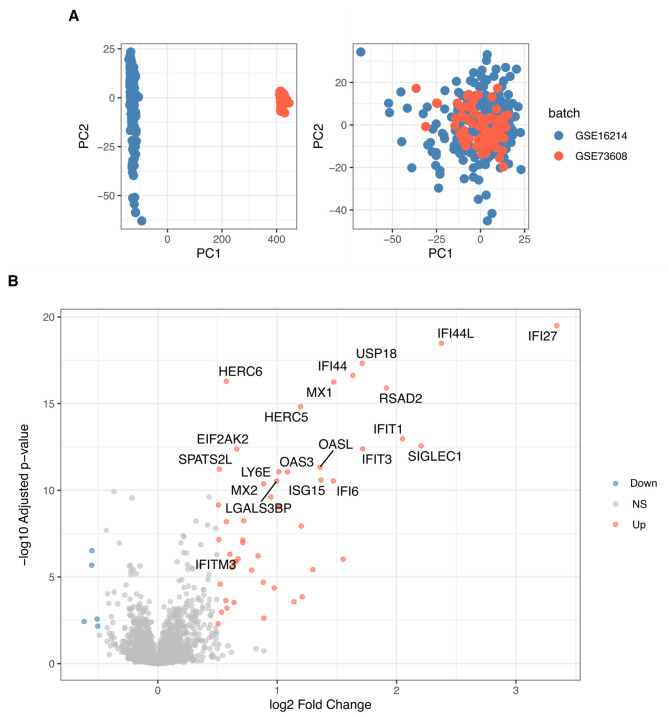
Transcriptomic effects of IFN-β treatment in PBMCs from MS patients. (**A**) PCA of the combined PBMC transcriptomic datasets GSE16214 and GSE73608 before (left) and after (right) ComBat batch-effect correction, showing improved integration of samples across datasets. Colors indicate dataset origin. (**B**) Volcano plot showing differentially expressed genes between IFN-β–treated and untreated patients. Differential expression analysis identified 55 IFN-β–responsive genes (FDR-adjusted *p* < 0.05 and |log2FC| > 0.5). Upregulated genes are shown in red, downregulated genes in blue, and non-significant genes in gray.

Datasets GSE16214 and GSE73608 were integrated using ComBat to correct for batch effects and platform-related variability. Differential expression analysis was performed within this harmonized space to identify PBMC genes modulated by IFN-β treatment. We applied ComBat correction and principal component analysis (PCA), revealing a strong batch effect prior to correction, which was effectively mitigated after ComBat adjustment while preserving treatment-related variance (Figure 1A). Differential expression analysis between IFN-β treated and untreated patients identified 55 genes as IFN-β-responsive genes in the PBMCs of MS patients, with several canonical ISGs, including Interferon Alpha-Inducible Protein 27 (IFI27), Interferon Induced Protein 44 Like (IFI44L), Ubiquitin-Specific Peptidase 18 (USP18), MX1, and Interferon-Stimulated Gene 15 (ISG15), among the most significantly upregulated transcripts (Supplementary Table S2a and Figure 1B).

**Figure 2:**
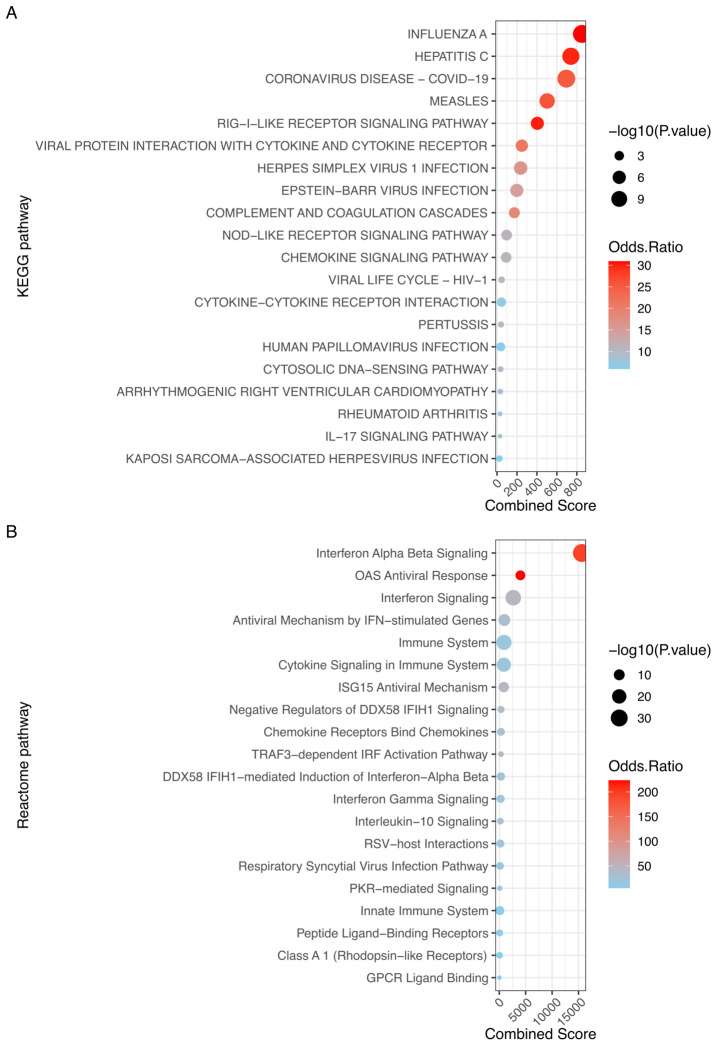
Functional enrichment analysis of IFN-β-responsive genes in PBMCs from MS patients. (**A**) KEGG pathway enrichment analysis and (**B**) Reactome pathway enrichment analysis, highlighting enrichment of IFN-related signaling pathways and antiviral response pathways. Dot size represents enrichment significance (−log10 adjusted *p*-value), whereas color intensity indicates the odds ratio. The x-axis reports the combined enrichment score.

KEGG pathway enrichment analysis on the identified 55 genes resulted in a significant overrepresentation of antiviral and innate immune pathways among IFN-β-responsive genes. The most enriched pathways included, Influenza A, Hepatitis C, Coronavirus disease–COVID-19, RIG-I-like receptor signaling pathway, and viral protein interaction with cytokine and cytokine receptor, herpes simplex virus 1 and EBV infection, consistent with activation of interferon-driven antiviral programs (Figure 2A). Reactome pathway enrichment analysis highlighted strong enrichment of interferon-related signaling pathways, including Interferon Alpha/Beta Signaling, OAS antiviral response, Interferon Signaling, and Antiviral Mechanism by IFN-stimulated Genes (Figure 2B). Gene Ontology (GO) Biological Process enrichment analysis further confirmed the strong activation of interferon-mediated antiviral and innate immune responses in IFN-β–treated patients. Among the most significantly enriched biological processes were IL-27-mediated signaling pathway, negative regulation of viral genome replication, antiviral innate immune response, and defense response to virus (Supplementary Figure S1).

Collectively, these enrichment analyses indicated that IFN-β treatment is associated with the activation of a coordinated interferon-driven antiviral transcriptional program and immune signaling network in PBMCs from MS patients. The complete lists of significantly enriched pathways are reported in Supplementary Table S2b–d.

### 2.2. Machine Learning Classification of IFN-β Response Reveals IFITM3 as MS-Linked eQTL Gene

**Figure 3:**
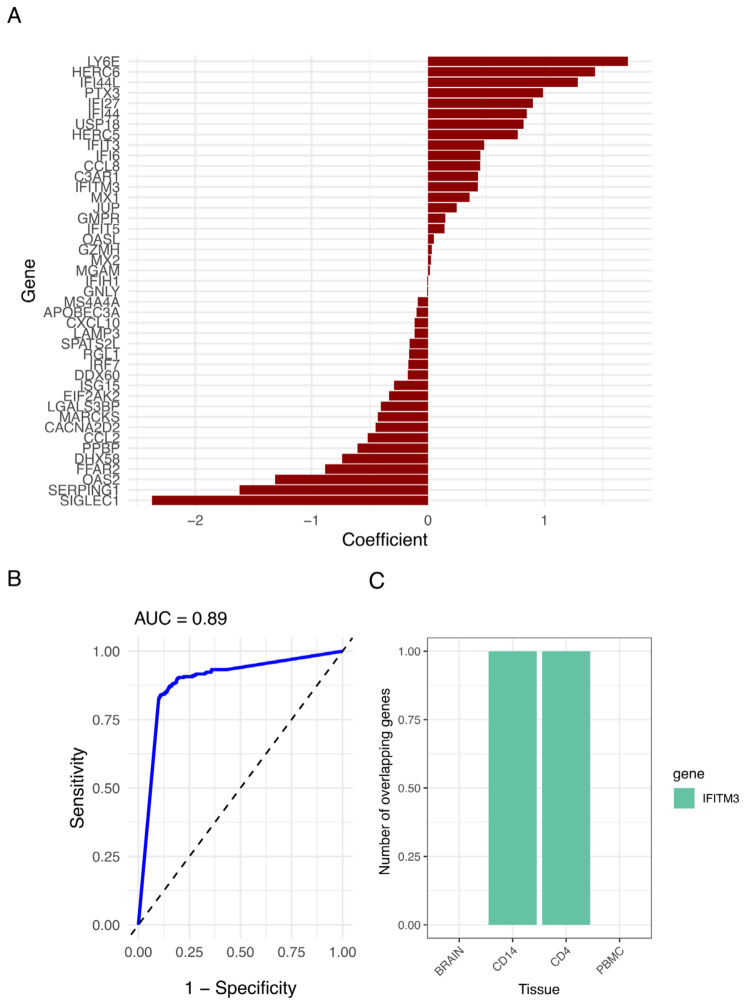
(**A**–**C**) Feature importance and model performance of the predictive gene signature. (**A**) Coefficient weights of selected genes in the predictive model, ranked by magnitude, highlighting positively and negatively associated transcripts contributing to the classification outcome. (**B**) ROC curve evaluating model discrimination performance, with an area under the curve (AUC) of 0.89, indicating strong ability to distinguish between classes. (**C**) Overlap between the eQTL gene lists retrieved from MS genomic map [34] and the IFN-genes included in panel (**A**) The analysis identifies IFITM3 as one of the model-selected genes carrying a significant regulatory variant; its associated SNP passes the FDR significance threshold specifically in naïve CD4^+^ T cells and monocytes.

Then, we applied a machine learning approach to evaluate the ability of the IFN-β-responsive transcriptional signature to discriminate IFN-β-treated patients from untreated individuals. An Elastic Net regression model was trained on the integrated and batch-corrected PBMC-derived dataset generated through ComBat harmonization of GSE16214 and GSE73608 using the 55 IFN-β–modulated genes, and subsequently validated in the independent whole-blood cohort GSE41850. Elastic Net regression retained 43 of the 55 genes, which are displayed in a coefficient bar plot illustrating their relative contribution to sample classification (Figure 3A). Overall, the model correctly classified samples, achieving an accuracy of 85.4%, with high sensitivity (94.0%) and moderate specificity (71.5%). These results demonstrate a strong capacity to identify IFN-β-treated patients while maintaining adequate discrimination of untreated individuals. Model performance was further evaluated using confusion matrix-derived metrics (Supplementary Table S3) and receiver operating characteristic (ROC) curve analysis (Figure 3B). Importantly, despite the validation cohort being generated from whole blood rather than PBMCs, the preserved predictive performance supports the robustness and cross-tissue reproducibility of the identified IFN-β transcriptional signature across blood-derived specimens.

Finally, we queried the IMSGC eQTL dataset of peripheral immune cells and microglia [34] (Supplementary Tables S4a–c) to determine whether Elastic Net-selected genes overlapped with MS susceptibility loci. IFITM3 emerged as the sole overlapping gene among the 200 non-MHC autosomal MS-associated genes (Supplementary Table S4a). Its associated SNP, rs35218683, exceeded the 5% FDR threshold and showed significant eQTL associations in naïve CD4^+^ T cells and monocytes (Figure 3C and Supplementary Table S4b). Notably, the protective C allele was associated with increased IFITM3 expression, whereas the MS risk T allele correlated with reduced IFITM3 expression in both cell types (Supplementary Table S4c). Interestingly, this SNP is located in a region proximal to the transcription start site (TSS) of IFITM3, encodes for an antiviral effector protein that restricts viral entry and replication within host cells, strengthening a potential link between antiviral immune mechanisms and MS pathogenesis [35].

## 3. Discussion

MS is a highly heterogeneous disease from a pathophysiological standpoint, encountering several distinct inflammatory and immune-mediated processes. This complexity also reflects in the clinical diversity of MS patients with vast epidemiology and clinical phenotypes and in the variability of therapeutic responses. From a clinical perspective, IFN-β remains a valuable therapeutic option for de-escalation strategies and for older patients, in whom safety considerations may outweigh the need for high-efficacy immunosuppression [6]. To date, the exact mechanism underlying the DMTs’ response variability in MS patients is unknown. Moreover, no specific biological markers are available. Identifying reliable biomarkers of treatment response may deepen understanding of IFN-β mechanisms of action and facilitate the transition toward a precision medicine-based therapeutic model.

Of interest, recent evidence indicates that the Type I Interferon pathway is connected with viral response and EBV [31]. To date, EBV represents the most recognized risk factor for MS, with the risk of MS increasing 32-fold after infection with EBV but not increasing after infection with other viruses. EBV is associated with higher risk compared to any other known risk factor for MS and this suggests EBV as the leading cause of MS [32].

Many ISGs have been identified over the years that could help monitor treatment response [25,26,33]. Among potential biomarkers, myxovirus resistance protein A (MxA) and other promising molecules are currently under investigation [36,37]. However, baseline expression levels of these genes in whole blood were not predictive of IFN-β response.

On these bases, we performed an integrative analysis of the gene expression profile of PBMC in MS patients treated with IFN-β, identifying a set of interferon-modulated genes. We identified 43 ISGs with a consistent overlap with the literature [24,25,38,39,40]. HECT and RLD Domain Containing E3 Ubiquitin Protein Ligase 5 (HERC5), IFI44L, Interferon-Induced Protein with Tetratricopeptide Repeats 1 (IFIT1), MX1, Radical S-Adenosyl Methionine Domain Containing 2 (RSAD2), Sialic Acid Binding Ig-Like Lectin 1 (SIGLEC1) [24,25], Eukaryotic Translation Initiation Factor 2 Alpha Kinase 2 (EIF2AK2), HERC6, IFI6, IFIT3, Lectin, Galactoside-Binding Soluble 3 Binding Protein (LGALS3BP), 2′-5′-Oligoadenylate Synthetase-Like protein (OASL) [25] and IFI27, USP18 [24] have been indicated as ISGs in previous studies of transcriptional response to IFN-β in MS patients. Moreover, some of the genes identified in our study including C-X-C Motif Chemokine Ligand 10 (CXCL10), IFIT2, ISG15, OAS3, and XAF1 are currently under investigation for their potential as blood biomarkers for IFN-β therapy in MS [38,41,42,43]. EIF2AK2 has been detected as one of the deregulated molecules in MS using a system biology approach [44]. Its physical interaction with STAT1 and ISG15, and its downstream regulation of IRF, have been demonstrated. The ISG15 and IFIT2:IFIT3 complex has been indicated in the same study. Previous research reported that IFIT3 was able to promote microglia polarization towards the pro-inflammatory M1 phenotype, therefore contributing to MS progression [45]. Recent quantitative proteomic studies detected DEAD-Box Helicase 60 (DDX60), together with other proteins related to antiviral pathways (e.g., MX1, OAS, OAS3, and OASL), as upregulated during Th17 differentiation, suggesting a role for IFN signaling in Th17 polarization [46].

In the second part of the analysis, we identified IFITM3 as a putative molecular mediator of MS disease. Among the 200 non-MHC autosomal MS-associated genes, IFITM3 emerged as the only gene overlapping with our IFN-β–modulated signature. The regulatory variant rs35218683, located near the IFITM3 transcription start site, showed significant eQTL effects specifically in naïve CD4^+^ T cells and monocytes, indicating a cell-type-restricted regulatory architecture [34].

Given that IFITM3 encodes a key antiviral effector that restricts viral entry and replication, this genetic–transcriptional convergence supports the possibility that IFN-driven antiviral pathways intersect with MS susceptibility mechanisms [35]. While few downstream effector ISGs have been de-linked to viral restriction in humans, IFITM3 has emerged as a critical exception. Clinical data indicates that a specific IFITM3 polymorphism, which exhibits impaired antiviral activity in vitro, is significantly overrepresented in patients experiencing severe or pandemic influenza requiring hospitalization. Review of the literature indicates that IFITM3 SNPs are primarily associated with increased viral disease in infections with emergent influenza viruses, such as the 2009 H1N1 pandemic virus and zoonotic H7N9 virus. Similarly, IFITM3 SNPs are reported to be risk factors for increased severity in other emergent infections, including SARS-CoV-2, Hantaan virus, and HIV [47,48].

Additionally, this gene was identified as a candidate biomarker exhibiting differential expression across distinct MS subtypes [45,49]. Lastly, variability of IFITM3 was hypothesized to preventing its antiviral action, contributing to the entry of viral components in normal-appearing white matter cells [50]. Given the potential role of IFITM3 in viral infections, investigating its involvement in EBV infection would be of considerable interest; however, to our knowledge, no evidence in the relation IFITM3-EBV-MS has been reported to date.

The presence of an MS-associated regulatory variant in a gene robustly induced by IFN-β treatment suggests a potential point of interaction between inherited risk and therapeutic response, highlighting IFITM3 as a biologically meaningful node linking antiviral immunity and MS pathogenesis.

IFITM3 was among the top 15 weighted predictors within the Elastic Net classifier; it displayed a positive coefficient consistent with IFN-β-responsive transcriptional activation. Collectively, the genes with a positive coefficient define a coordinated interferon-driven immune network, including classical ISGs, and are characterized by antiviral defense and innate immune activation. In this context, IFITM3 emerged as the only gene converging across differential expression, Elastic Net feature selection, and MS susceptibility eQTL analyses, thereby strengthening its biological relevance.

A limitation of our study is the lack of data on neutralizing anti-IFN-β antibodies (Nabs), which would have been highly informative, as their presence is a major factor influencing the therapeutic efficacy of IFN-β. NAbs bind to the IFN-β molecule, prevent its interaction with IFNAR, and block downstream IFN-β signaling, and they can develop in some patients treated with IFN-β [38]. Assessing NAbs development could therefore provide an additional parameter for stratifying patients based on gene expression profiles.

Another limitation of this study is that the analyses were focused on identifying transcriptional differences between IFN-β-treated and untreated MS patients, without the availability of clinical response or longitudinal outcome data. Therefore, the identified signature should be interpreted as associated with IFN-β treatment rather than as a predictive biomarker of therapeutic response. In addition, transcriptomic findings are based on integrative bioinformatics analyses and still require experimental validation in independent cohorts and relevant immune cell subsets at the transcript and protein levels.

Furthermore, the association between IFITM3 and MS susceptibility was derived from a single published eQTL resource and was not independently replicated using additional GWAS, eQTL, or transcriptomic datasets. Although the convergence of differential expression, Elastic Net feature selection, immune cell-specific eQTL associations, and independent protein-level observations support the biological plausibility of IFITM3 involvement, the current evidence remains primarily associative. Therefore, IFITM3 should be regarded as a candidate gene linking IFN-β response pathways to MS susceptibility, and independent computational and experimental validation will be required to establish a causal mechanistic role.

Finally, this study delineated a distinct transcriptional signature composed of 43 genes modulated by IFN-β therapy in MS. This comprehensive profiling not only underscores the immunomodulatory impact of IFN-β at the molecular level but also enabled the identification of IFITM3 as a molecular target of IFN-β and a putative molecular mediator of MS disease. This result provides new insights into the mechanisms underlying IFN-β-mediated immune regulation, suggesting their potential utility as biomarker discovery, MS pathogenesis, and therapeutic relevance.

## 4. Materials and Methods

### 4.1. Transcriptome Analysis of IFN-β-Treated MS Patients

Three publicly available transcriptomic datasets were analyzed to characterize the molecular effects of IFN-β treatment in multiple sclerosis (MS): GSE16214, GSE73608, and GSE41850 (Supplementary Table S1). Raw expression matrices and sample metadata were retrieved using the GEOquery package version 2.74.0. Sample metadata were harmonized to derive a unified binary treatment variable (“treated” vs. “untreated”). Samples treated with glatiramer acetate or lacking interpretable treatment information were excluded. Probe-level intensities were annotated with gene symbols, and genes represented by multiple probes were collapsed by averaging their expression values. Only genes shared across both platforms were retained for integration.

To integrate PBMC datasets (GSE16214, GSE73608), we applied ComBat (sva package version 3.54.0) using a model matrix including the treatment variable to preserve biological signal. PCA was performed before and after correction to assess batch-effect removal and sample mixing across datasets. The resulting ComBat-adjusted expression matrix was used for all downstream PBMC-based analyses. Differential expression between IFN-β-treated and untreated PBMC samples was performed using the Linear Models for Microarray Analysis (Limma) package version 3.62.2. Linear models were fitted on the ComBat-corrected matrix, followed by empirical Bayes moderation. Genes were considered differentially expressed if they satisfied: FDR-adjusted *p*-value < 0.05 and |log_2_ fold change| > 0.5.

### 4.2. Functional Enrichment Analysis of IFN-β-Responsive Genes

Functional enrichment of the 55 IFN-β-responsive genes was performed using enrichR across three databases: GO Biological Process 2025; KEGG 2026 and Reactome Pathways 2024. Enrichment significance was evaluated using combined scores, odds ratios, and adjusted *p*-values. The top 20 enriched pathways per database were visualized using dot plots generated with ggplot2. Results highlighted strong activation of antiviral and IFN-mediated immune pathways.

### 4.3. Machine Learning Classification of IFN-β Treatment Status

The ComBat-integrated PBMC dataset served as the training set. Only the 55 IFN-β–responsive genes were retained as predictors. The independent validation set consisted of whole-blood samples from GSE41850. Gene symbols were extracted from the platform annotation, harmonized, and aligned to the PBMC-derived feature space. Expression values were log_2_-transformed and scaled using the mean and standard deviation of the training set to ensure cross-dataset comparability. Penalized logistic regression models were trained using glmnet: Elastic Net (α = 0.5). Ten-fold cross-validation (type.measure = “auc”) was used to select the optimal λ. Coefficients at λ_min were extracted to identify genes retained by each model. Model performance was assessed on all test sets using ROC/AUC and confusion matrices. The Elastic Net model retained 43 genes, which were used for downstream interrogation of publicly available eQTL datasets.

### 4.4. eQTL Annotation of Elastic Net-Selected Genes

To investigate the genetic regulation of the IFN-β-responsive signature, we retrieved immune-cell and microglia eQTL data from the Supplementary Materials of the IMSGC study [34] (Supplementary Table S4a–c). We screened the eQTL datasets for genes identified by the Elastic Net model and evaluated their associated risk SNP–gene expression associations, retaining only significant eQTLs (FDR < 5%) across naïve CD4^+^ T cells, CD14^+^ monocytes, PBMCs, and brain tissue. IFITM3 emerged as the only overlapping gene across datasets. For each MS susceptibility variant, the published summary statistics included Spearman correlation coefficient (rho), significance level, and FDR status across CD4^+^ T cells, CD14^+^ monocytes, PBMCs, and brain tissue. Directionality was inferred from the sign of the reported Spearman correlation coefficients. Positive rho values indicate that the allele is associated with increased gene expression, whereas negative values indicate reduced expression. No additional eQTL modeling was performed in the present study.

### 4.5. Software

All the analyses have been performed in R version 4.4.2 (31 October 2024).

## Data Availability

The original contributions presented in this study are included in the article and its Supplementary Material. The code used for the analyses is publicly available at “https://github.com/AlessandroMaglione/IFNb-transcriptomics-MS (accessed on 25 May 2026)”.

## Supplementary Materials

The following supporting information can be downloaded at https://www.mdpi.com/article/10.3390/ijms27125329/s1.
