# Supplementary material for: Integrative Transcriptomics Uncovers IFN-β Signature and IFITM3 as Putative Molecular Mediator in MS
Source: Int J Mol Sci. 2026 Jun 12;27(12):5329. doi: 10.3390/ijms27125329 (PMC13299371; doi:10.3390/ijms27125329)
Supplement: Supplementary file 1 [file ijms-27-05329-s001.zip › Supplementary material-ijms-4195708_tableS1_S3_figureS1.pdf]

Supplementary material

This file include:

Supplementary Table S1. Composition of the cohorts included in the studies considered for the analysis of ISG in MS patients.

Supplementary Figure S1. Gene Ontology Biological Process enrichment analysis of IFN-β–responsive genes.

Supplementary Table S3. Confusion matrix of the Elastic Net model.

Supplementary Table S1. Composition of the cohorts included in the studies considered for the analysis of ISG in MS patients.

GEO accession ID	Experiment al platform	MS course	Sampling time	Number of samples	Sample type	Reference
**GSE16214**	Microarray - GPL570	CIS-RRMS	3 months	MS patients (n = 94 treated, n = 82 untreated)	PBMC	Ottoboni_2012 - PMID: 23019656
**GSE41850**	Microarray - GPL16209	CIS-RRMS-SPMS	0 - 1year - 2years	MS patients (n = 106 treated, n = 89 untreated),	Whole blood	Nickles_2013 - PMID: 23748426
**GSE73608**	Microarray – GPL571	SPMS	2 years	SPMS patients (n = 25 treated, n = 49 untreated)	PBMC	Gurevich_2015 - PMID: 26589141

Abbreviations. GEO: Gene Expression Omnibus, CIS: clinically isolated syndrome, RRMS: relapsing-remitting MS, MS: multiple sclerosis, SP: secondary progressive MS.

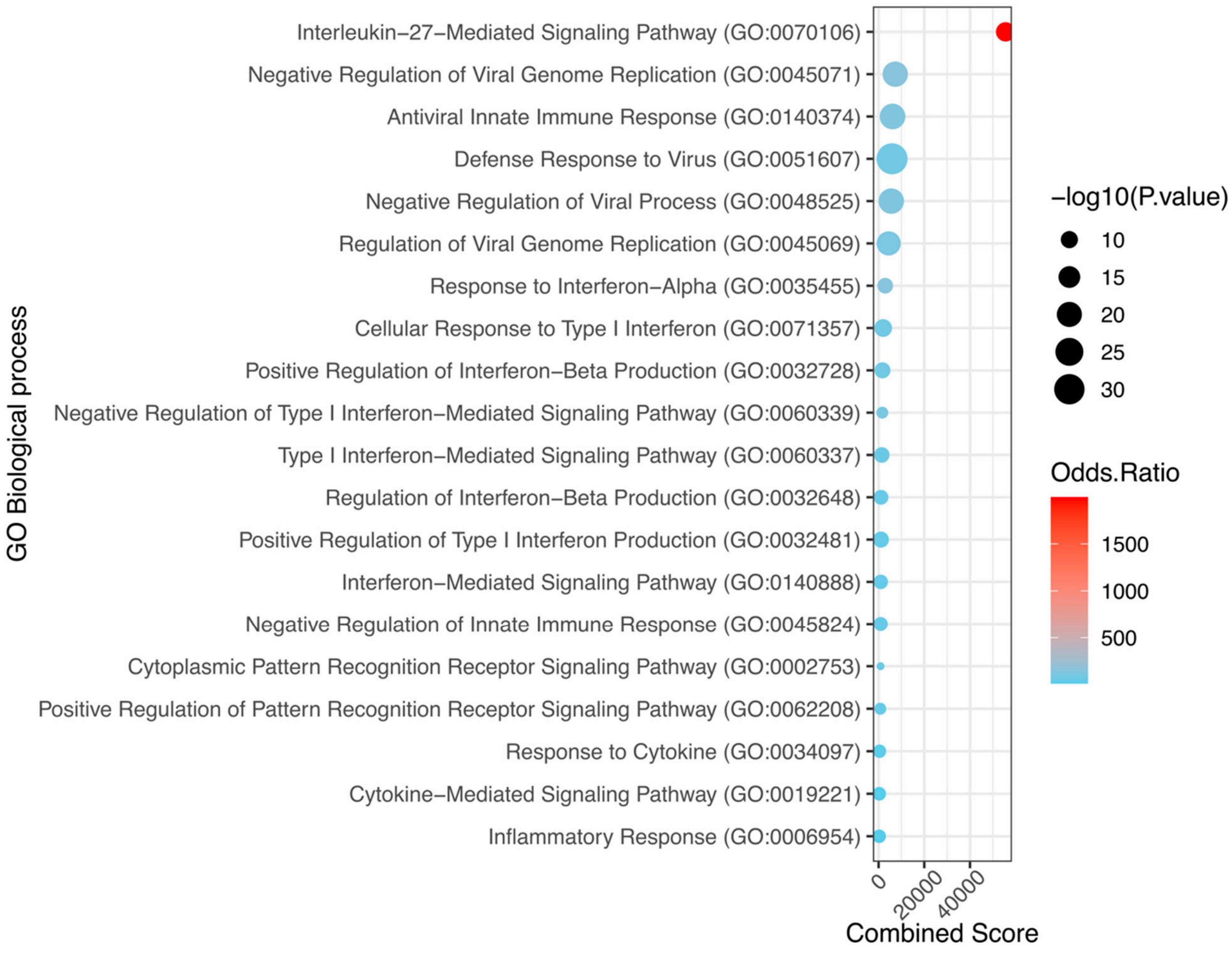

Supplementary Figure S1. Gene Ontology Biological Process enrichment analysis of IFN-β–responsive genes.

Dot plot showing significantly enriched GO Biological Process categories associated with IFN-β–responsive genes identified in PBMCs from MS patients. Dot size represents enrichment significance (−log10 adjusted *p*-value), color intensity indicates the odds ratio, and the x-axis reports the combined enrichment score.

Prediction	Reference	Freq
0	0	270
1	0	233
0	1	18
1	1	294

Supplementary Table S3. Confusion matrix of the Elastic Net model. Confusion matrix summarizing the classification performance of the Elastic Net model trained on the IFN-β–responsive gene signature. The table reports the number of correctly and incorrectly classified samples in the independent whole-blood validation cohort.
